# Application of Novel Short Tandem Repeat Typing for *Wickerhamomyces anomalus* Reveals Simultaneous Outbreaks within a Single Hospital

**DOI:** 10.3390/microorganisms11061525

**Published:** 2023-06-08

**Authors:** Bram Spruijtenburg, Shivaprakash M. Rudramurthy, Eelco F. J. Meijer, Merlijn H. I. van Haren, Harsimran Kaur, Arunaloke Chakrabarti, Jacques F. Meis, Theun de Groot

**Affiliations:** 1 Department of Medical Microbiology and Infectious Diseases, Canisius-Wilhelmina Hospital, 6532 SZ Nijmegen, The Netherlands; 2 Centre of Expertise in Mycology, Radboud University Medical Center/Canisius Wilhelmina Hospital, 6532 SZ Nijmegen, The Netherlands; 3 Department of Medical Microbiology, Postgraduate Institute of Medical Education and Research, Chandigarh 160012, India; 4 Department I of Internal Medicine, University of Cologne, Excellence Center for Medical Mycology, 50931 Cologne, Germany

**Keywords:** *Wickerhamomyces anomalus*, *Candida pelliculosa*, short tandem repeats, genotyping, whole-genome sequencing, antifungal susceptibility testing, outbreak investigation

## Abstract

*Wickerhamomyces anomalus,* previously known as *Candida pelliculosa,* occasionally causes candidemia in humans, primarily infecting neonates, and infants. The mortality rate of these invasive infections is high, and isolates with a reduced susceptibility to fluconazole have been reported. *W. anomalus* outbreaks are regularly reported in healthcare facilities, especially in neonatal intensive care units (NICUs). In order to rapidly genotype isolates with a high-resolution, we developed and applied a short tandem repeat (STR) typing scheme for *W. anomalus*. Six STR markers were selected and amplified in two multiplex PCRs, M3 and M6, respectively. In total, 90 *W. anomalus* isolates were typed, leading to the identification of 38 different genotypes. Four large clusters were found, unveiling simultaneous outbreak events spread across multiple units within the same hospital. STR typing results of 11 isolates were compared to whole-genome sequencing (WGS) single nucleotide polymorphism (SNP) calling, and the identified genotypic relationships were highly concordant. We performed antifungal susceptibility testing of these isolates, and a reduced susceptibility to fluconazole was found for two (2.3%) isolates. *ERG11* genes of these two isolates were examined using WGS data, which revealed a novel I469L substitution in one isolate. By constructing a homology model for *W. anomalus ERG11p*, the substitution was found in close proximity to the fluconazole binding site. In summary, we showed multiple *W. anomalus* outbreak events by applying a novel STR genotyping scheme.

## 1. Introduction

Fungal diseases affect over a billion people, causing an estimated 1.5 million deaths every year, which appears to be increasing [1,2]. *Candida* species are one of the leading causative agents of invasive fungal infections, with an attributable high mortality rate primarily affecting those with underlying hematological or oncological malignancies, intensive care unit admission, recent major surgery, and/or central venous catheter placement [3,4,5]. To date, *Candida albicans* is the single most common cause of invasive candidiasis [6,7], although the share of non-*C. albicans* spp. yeast infections is increasing, and in some countries, it is surpassing *C. albicans* [3,7,8]. The emergence of new resistant species, including but not limited to those with acquired resistance such as (pan-)resistant *C. auris,* and azole-resistant *C. tropicalis* and *C. parapsilosis,* poses an additional and potentially even bigger threat [9,10,11,12]. Another emerging species is *Wickerhamomyces anomalus,* previously known as *Candida pelliculosa, Pichia anomala* and *Hansenula anomala*. It is a rare but increasingly reported causative agent of nosocomial fungemia outbreaks, preponderantly in low-birth-weight (LBW) infants and occasionally in immunocompromised patients [13,14,15,16,17,18]. In a multicentric study of pediatric candidemia in ICU settings in India, *W. anomalus* was reported as the third most common agent, especially in neonates [19].

*W. anomalus* is a diploid ubiquitous yeast, found in varying environments including fruits, trees, soil, waste water, marine environments and even the guts of insects [20,21]. Surprisingly, *W. anomalus* is highly tolerant to environmental stresses. It can develop biofilms and is adapted to a wide range of growth conditions, including temperature (3–37 °C) and pH value (2–12), and has even demonstrated unusual antimicrobial activity against other yeasts, filamentous fungi, and bacteria [22,23]. Its unique properties have made this yeast biotechnologically interesting, leading to its current use in food, environmental, industrial, and medical applications [21].

Taxonomical (mainly genus) changes in the field of mycology have recently drastically changed the naming of *Candida* spp., mostly due to the widespread application of molecular technologies in taxonomy, allowing for corrections in the existing system [24]. However, these novel names may not yet be globally implemented and of semantic consideration; here, *W. anomalus* is used instead of *Candida pelliculosa, Pichia anomala*, or *Hansenula anomala* [25]. MALDI-TOF MS using an updated valid database and molecular diagnostics like ITS sequencing or whole genome sequencing (WGS) allow accurate identification of this species, and molecular diagnostics such as ITS sequencing or whole-genome sequencing (WGS) can be used to verify species identification. Correct species identification using biochemical tests or other commercial yeast diagnostic kits can be challenging [26].

Globally, antifungal-resistant isolates are emerging, especially against azoles [5,7,27,28]. Azole resistance can either be intrinsic, for example, in *Candida krusei* or *Candida glabrata*, or can be acquired in species such as *Candida tropicalis* or *C. parapsilosis* [26,27]. Acquired fluconazole resistance in *Candida* species is often caused by missense mutations in *ERG11,* which decrease fluconazole binding affinity to *ERG11* and therefore maintain membrane integrity [29,30]. Other resistance mechanisms include missense mutations in other *ERG* genes, or mutations in transcription factors which induce upregulation of multidrug efflux pumps [31]. The emergence of multiple *W. anomalus* isolates with a decreased susceptibility to fluconazole have been recently reported, although resistance mechanisms have not been described [13,32,33].

*W. anomalus* hospital outbreaks have been observed, predominantly in neonatal intensive care units (NICUs) or pediatric wards, often related to contaminated foods or horizontal spread from healthcare personnel due to insufficient infection prevention and control measures [13,18,34,35,36,37]. When *W. anomalus* is identified in multiple patients in a single healthcare facility, rapid and high-resolution genotyping is essential. With an adequate genotyping method, cross-transmission can be investigated, the source of infection can be localized, and transmission may be controlled. *W. anomalus* isolates have been typed with several methods, including multilocus enzyme electrophoresis (MLEE), electrophoretic karyotyping (EK), inter-repeat PCR (IR-PCR) and random amplified polymorphic DNA (RAPD) assays [18,32]. Many of these methods lack the necessary discriminatory power and are lengthy and laborious to perform. Therefore, these methods have largely been rendered futile due to recently developed PCR and sequencing-based methods. WGS has the best resolution, is reliable and has excellent reproducibility and discriminatory power; however, it is costly and has a long turn-around time, the latter being undesirable in possible outbreak settings.

An appealing alternative genotyping method with a high resolution is short tandem repeat (STR) or microsatellite genotyping, which is set up for species separately and has proved to be highly reproducible, rapid and cost-effective while being easy to execute [38,39,40,41]. STR genotyping results are straightforward, and they are suitable to be exchanged between laboratories. However, this method might not be amenable in resource limited labs due to the costs. Here, we applied a novel STR typing scheme to *W. anomalus* isolates, mainly retrieved from a large tertiary care center in India. After having validated the STR genotyping by WGS SNP analysis, we describe multiple large outbreak events within a single hospital.

## 2. Materials and Methods

### 2.1. Isolates

In total, 90 clinical *W. anomalus* isolates were used in this study (Table S1). In addition, 14 other *Candida*, *Cryptococcus*, *Saccharomyces* and *Kodameae* species were included to test the specificity of the novel typing scheme (Table S2). Isolates were stored according to standard procedures at −80 °C. All isolates were identified by matrix-assisted laser desorption ionization–time-of-flight (MALDI-TOF) mass spectrometry as previously described [42]. This study was approved by the ethics committee of the Postgraduate Institute of Medical Education and Research (PGIMER).

### 2.2. Culture and DNA Extraction

Isolates were taken from storage at −80 °C and grown on Sabouraud agar plates (Oxoid, Hampshire, UK) at 30 °C. For STR genotyping and WGS, a single colony of each isolate was resuspended in 400 µL MagNA Pure bacteria lysis buffer and MagNA Lyser green beads. These were mechanically lysed for 30 s at 6500 rpm using the MagNA Lyser system (all Roche Diagnostics GmbH, Mannheim, Germany). DNA was extracted and purified with the MagNA Pure 96 instrument and the MagNA Pure DNA and Viral NA Small Volume Kit (Roche Diagnostics), following the manufacturer’s instruction. For WGS, all samples were subsequently treated with RNase (Sigma-Aldrich, Burlington, MA, USA) at a final concentration of 5 µg/µL for one hour at room temperature, after which DNA was extracted and purified as described above. Purified DNA was measured with a Qubit 3.0 Fluorometer (Thermo Fisher Scientific, Waltham, MA, USA) using the double-stranded DNA (dsDNA) high sensitivity option.

### 2.3. Identification of STR Loci

The *W. anomalus* reference genome KG16 (GCA_019321765.1) was downloaded from the NCBI database and uploaded to the Tandem Repeat Finder (https://tandem.bu.edu/trf/trf.html, accessed on 4 July 2022) [43]. STRs were identified using the advanced search option (alignment parameter, 2,7,7; minimum alignment score to report repeat, 90; maximum period size, 10; maximum tandem repeat array size, 2). Repeats with insertions or deletion, exhibiting a <90% perfect match to the repeat sequence or having a copy number of <10, were excluded. From the remaining STRs, three repeats with a period size of three nucleotides and three repeats with a period size of six nucleotides were selected based on their copy number.

### 2.4. Whole-Genome Sequencing

Genomic libraries were prepared and sequenced with the Illumina NovaSeq 6000 platform (Illumina, San Diego, CA, USA) with 2-by-150 bp paired-end-read mode at Eurofins Genomics (Ebersberg, Germany). Read data were uploaded to the Galaxy tool, FastQC was used to assess the quality of the read data, and no trimming was performed [44]. Read data were aligned against the *W. anomalus* reference genome KG16 (GCA_019321765.1) using BWA-MEM [45]. PCR duplicates were removed using RmDup, local realignment was performed with BamLeftAlign, and unpaired reads were removed with BAM filter. Mapped reads with a MAPQ score < 60 were removed. WGS alignments of the six selected STRs and flanking sequences were visually inspected using JBrowse v1.16.11 to identify variants in primer binding sites [46]. All raw read data generated in this study have been submitted to the National Center for Biotechnology Information’s Sequence Read Archive (BioProject ID: PRJNA851035).

### 2.5. Primer Design and PCR Genotyping

Primers were designed with Primer3Plus (https://www.bioinformatics.nl/cgi-bin/primer3plus/primer3plus.cgi, accessed on 12 July 2022), using default settings, except for primer size (minimum [Min], 19; optimum [Opt], 21; maximum [Max], 24), primer melting temperature (T_m_) (Min, 57.0; Opt, 59.0; Max, 62.0), Max-Poly-X (3) and GC clamp (1) [47]. Variants in STR flanking sequences were marked as excluded regions. Primers that formed no self- or cross-dimers with five or more nucleotides from the last seven nucleotides of the 3′ end of a primer according to the multiple-primer analyzer from Thermo Fisher Scientific were ordered via Eurogentec (Cologne, Germany). Multiplex PCR was performed on a thermocycler (Biometra, Westburg, Göttingen, Germany) using 1x FastStart *Taq* polymerase buffer without MgCl_2_ (Roche Diagnostics, Germany), deoxynucleotide triphosphates (dNTPs) (0.2 mM), MgCl_2_ (3 mM), forward (fwd) and reverse (rev) primers (0.1 to 0.5 µM), 1 U FastStart *Taq* polymerase (Roche Diagnostics, Germany) and isolated DNA. A thermal protocol of 10 min of denaturation at 95 °C followed by 30 cycles consisting of 30 s denaturation at 95 °C, 30 s of annealing at 60 °C and 1 min of extension at 72 °C with a final incubation step for 10 min at 72 °C was used for PCR amplification. PCR products were diluted 1:1000 in water, and 10 µL together with 0.12 µL of the Orange 600 DNA size standard (NimaGen, Nijmegen, The Netherlands) were incubated for 1 min at 95 °C and analyzed on a 3500 XL genetic analyzer (Applied Biosystems, Foster City, CA, USA).

### 2.6. Data Analysis

All copy numbers of the six STR markers were determined using GeneMapper 5 software (Applied Biosystems). For all markers, minus-A peaks, stutter peaks, those with an intensity of >50% below the highest peak, and bleed-through peaks were discarded. Subsequently, the size of alleles was rounded. Copy numbers for repeats were converted to a binary matrix: 1, if an isolate contained the allele, and 0, if it did not. Relatedness between isolates was analyzed using BioNumerics software v7.6.1 (Applied Maths NV, Sint-Martems-Latem, Belgium) via the unweighted pair group method with arithmetic means (UPGMA), using the multistate categorical similarity coefficient.

### 2.7. WGS SNP Calling

SNPs in all isolates were detected with Freebayes using the default settings except for allelic scope options (ignore indels, multiple nucleotide polymorphisms [MNPs], and complex events) [48]. Resulting SNPs in the VCF file with a read depth (DP) of <25, a quality (QUAL) of <100, an allele frequency (OA) of between 0.15 × depth (DP) and 0.45 x DP and an allele frequency of between 0.45 × DP and 0.9 × DP were removed. Previously, the WGS SNP calling pipeline was validated with a *Candida auris* WGS benchmark dataset [49,50]. Phylogenetic analysis was performed with VCF2PopTree, using the genetic drift algorithm, and a MEGA distance based matrix was developed [51]. The matrix was uploaded to MEGA11 v11.0.10, and a phylogenetic tree was generated using the neighbor-joining tree method [52].

### 2.8. Antifungal Susceptibility Testing (AFST)

The in vitro minimum inhibitory concentrations (MIC) against azoles, amphotericin B and echinocandin drugs were determined according to CLSI microbroth dilution M27 standard [53]. In short, isolates were incubated at a concentration of 1 × 10^3^–5 × 10^3^ CFU/mL in RPMI medium. MIC values were read after 24 h of incubation at 35 °C as the lowest antifungal concentration with a 50% growth reduction when compared to the growth control, except for amphotericin B, for which a 100% growth reduction was used. Epidemiological cutoff values (ECVs) were implemented according to the CLSI M57S guideline to identify non-wildtype strains [54]. Applied ECVs are as follows: for amphotericin, B 1 mg/L; for fluconazole, 8 mg/L; for itraconazole, 1 mg/L; for micafungin, 0.12 mg/L; and for voriconazole, 0.25 mg/L.

### 2.9. Investigation Resistance-Associated Genes

Azole resistance-associated gene sequences of *ERG11* (NC_032093.1), *UPC2* (JX494823.1) and *ERG3* (AF069752.1) in *C. albicans* were identified in the *W. anomalus* KG16 genome using Nucleotide BLAST (https://blast.ncbi.nlm.nih.gov/Blast.cgi, accessed on 17 August 2022). Genes were visually inspected for homozygous substitutions solely present in fluconazole resistant isolates with JBrowse [46]. The coding sequence of *ERG11* determined in this study of isolate 10-11-09-22 was deposited in GenBank under the accession number ON805829. The *ERG11p* homology model was constructed with SWISS-MODEL (https://swissmodel.expasy.org/interactive, accessed on 17 August 2022) by using the template *Saccharomyces cerevisiae CYP51* (Lanosterol 14-alpha demethylase G73R mutant complexed with fluconazole (5ese.1.A) [55]. The predicted *ERG11* protein model was visually inspected to ensure that all major P450 structural motifs (I-helix, FG loop, and the substrate access channel) were intact. The resulting PDB file was uploaded to PyMOL v2.0, and fluconazole and the I469 amino acid were marked [56].

## 3. Results

### 3.1. Development and Application of W. anomalus STR Genotyping

**Figure 1:**
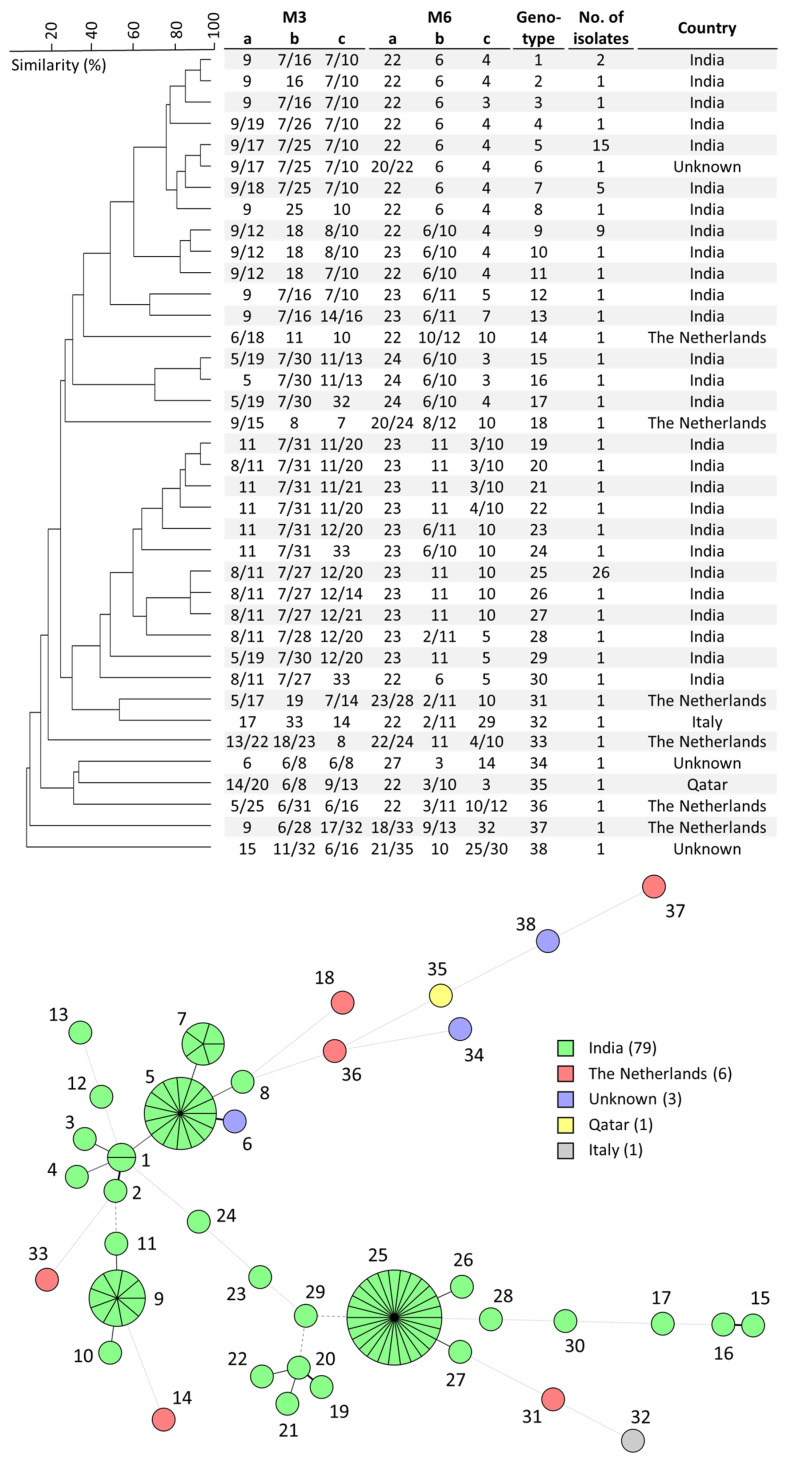
Cluster analysis of all *W. anomalus* isolates used in this study (**top**). Branch lengths indicate relatedness according to microsatellite alleles. The UPGMA dendrogram was generated with BioNumerics v7.5. Minimum-spanning tree of 90 *W. anomalus* isolates (**bottom**). Branch lengths indicate the similarity between isolates with thick solid lines (variation in one allele), thin solid lines (variation in two alleles), thin dashed lines (variation in three alleles) and thin dotted lines (variation in four or more alleles). Number of isolates per country is shown in the color key.

With the use of Tandem Repeat Finder, repeats in the *W. anomalus* reference strain KG16 were identified. Subsequently, a selection was made of three trinucleotide and three hexanucleotide repeats with the highest copy numbers (Table 1). WGS read data from five *W. anomalus* isolates were mapped against the KG16 genome. Flanking sequences of the selected tandem repeats were visualized to identify conserved regions, present in all five isolates. Primers were designed on these conserved regions in proximity to the repeat, screened for potential cross-dimer formation and coupled to fluorescent probes. The three trinucleotide and three hexanucleotide repeats were combined in two PCR multiplex reactions, M3 and M6, respectively, and optimal primer concentrations were determined (Table 1). Both multiplex PCRs were then applied to 90 clinical *W. anomalus* isolates, previously identified by MALDI-TOF (Table S1). All Indian isolates originated from the same hospital and were collected between 2018 and 2019 from neonatal and pediatric wards and ICUs. Each isolate exhibited one or two alleles per marker, which was expected because *W. anomalus* is a diploid species. The genotyping of all 90 *W. anomalus* isolates resulted in the identification of 38 different genotypes with four large clusters, consisting of between 5 and, at most, 26 isolates (Figure 1). The dates of fungemia were partially known for these closely related isolates, revealing that these outbreaks occurred in 2019 (Figure S1). In the three clusters with known information regarding involved hospital units, isolates originated from more than one hospital unit. Isolates from the Netherlands, Qatar and Italy all exhibited unique genotypes. Genotypes 1 and 2, 5 and 6, 15 and 16, and 19 and 20 were highly related to each other according to STR typing, exhibiting a zygosity difference in only one marker (Figure 1).

For specificity testing, 14 related yeast species were typed by both multiplex PCRs (Table S2). This resulted in no PCR products after amplification for any of the tested species, indicating that the STR assay is specific for *W. anomalus*. The stability of the STR markers was tested by subcloning five colonies of isolates 10-03-01-56 and 08-32-01-30 for ten generations. None of the copy numbers were altered, indicating high genetic stability. For reproducibility testing of the STR typing, the two previously mentioned isolates were independently genotyped, five times, in five replicate experiments. This resulted in identical results for all STR markers in all experiments, demonstrating that the genotyping method is highly reproducible.

### 3.2. WGS SNP Analysis

**Figure 2:**
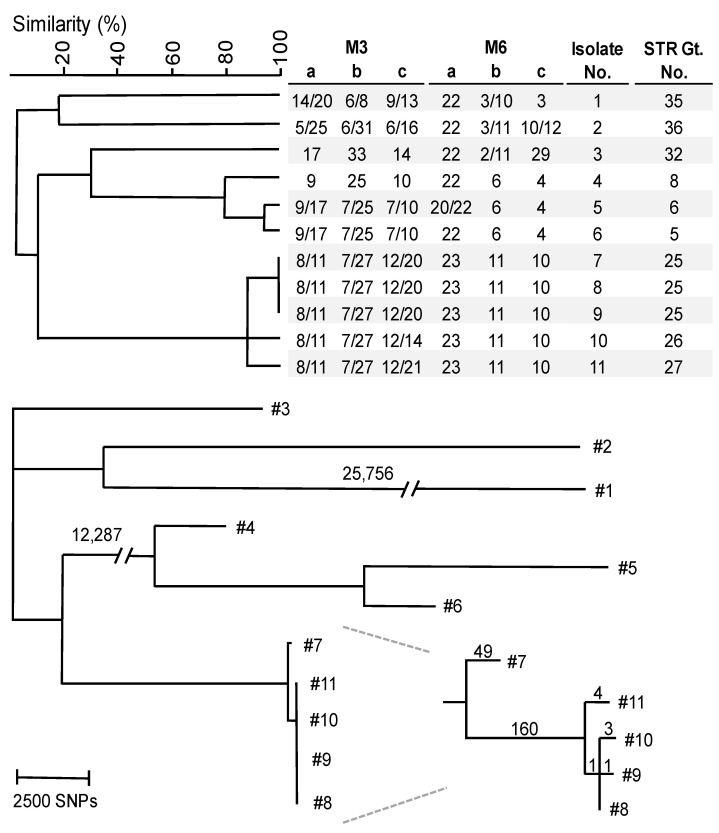
Comparison of genetic relatedness of 11 *W. anomalus* isolates by STR typing (**top**) and WGS SNP typing (**bottom**). The UPGMA dendrogram was generated with BioNumerics. The numbers above the branches of the phylogenetic tree indicate the number of SNPs. The tree was generated with MEGA11 using the neighbor-joining tree method. Isolates were renamed to provide a clear overview, #1: 10-03-01-56, #2: 10-11-09-22, #3: 10-09-05-80, #4: 08-32-01-29, #5: 10-04-08-74, #6: 08-32-01-15, #7: 08-32-01-05, #8: 08-32-01-14, #9: 08-32-01-07, #10: 08-32-01-02 and #11: 08-32-01-30.

In order to validate genotypical relatedness according to STR typing, 11 isolates were submitted for WGS. Reads from each strain were mapped to the KG16 reference genome, and SNPs between isolates were called with Freebayes (Figure 2). Five isolates from India, highly related or identical according to STR typing, clustered in one group of four isolates with a difference of eight SNPs at most, which was distinguished by 210 SNPs from the remaining isolate. The examination of these 210 SNPs with JBrowse showed that 203 of these SNPs were heterozygous and located in one part of a chromosome of approximately 400 kbp. Isolates with a difference of at least 10,000 SNPs compared to other isolates all demonstrated unique STR genotypes. Copy numbers of repeats with a total length below 140 bp were all confirmed visually in silico for sequenced isolates.

### 3.3. AFST and Resistance-Associated Genes Investigation

**Figure 3:**
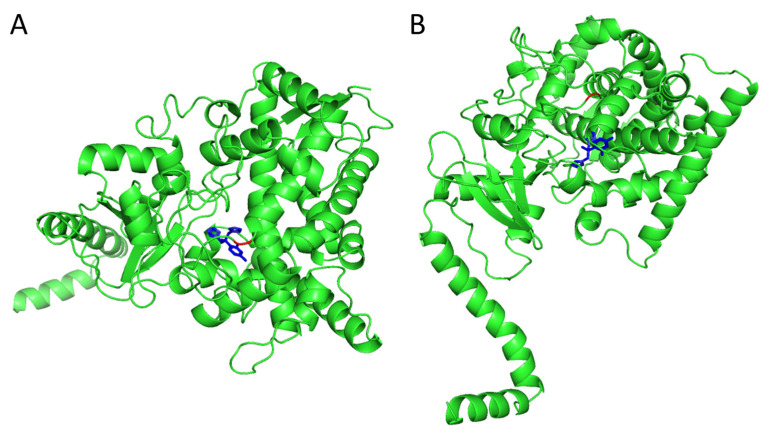
*W. anomalus* homology model of ERG11 constructed with SWISS-MODEL, top view (**A**) and rear view (**B**). Amino-acids, fluconazole and the I469 position are shown in green, blue and red, respectively.

AFST according to microbroth dilution was performed on 87 isolates against several azoles, amphotericin B and two echinocandins, and ECVs according to the CLSI M57S guideline were used to identify non-wildtype strains (Table S1). Only wildtype strains against micafungin were found, while some non-wildtype strains for amphotericin B, fluconazole, itraconazole and voriconazole were identified (Table 2). According to the CLSI ECVs, six isolates were non-wildtype (≥1 mg/L) to amphotericin B. Only two isolates (10-03-01-56 and 10-11-09-22) exhibited non-wildtype fluconazole MICs (8 and 16 mg/L) and originated from Qatar and the Netherlands, respectively. To search for a potential resistance mechanism, azole resistance-associated genes were located and visually inspected in the *W. anomalus* genome. The *W. anomalus ERG11* gene was located and subsequently aligned to *C. albicans ERG11* (Figure S2). Isolate 10-11-09-22 harbored an I469L substitution, while no substitutions were found in the other fluconazole non-susceptible isolate. The *ERG11* I469L substitution was located in a hotspot region of *C. albicans*. To investigate the potential impact of this substitution on fluconazole resistance, an *ERG11* homology model was constructed, based on a *Saccharomyces cerevisiae CYP51* template with a 70.65% sequence identify and a MolProbity Score of 1.14 (Figure 3). The I469 position is located in close proximity to the heme-binding site, which is also used by azole derivatives. No substitutions solely present in fluconazole resistant isolates were found for *UPC2* and *ERG3*. With nucleotide blast, efflux transcription factors *TAC1* and *MRR1* could not be identified based on orthologous *C. albicans* genes.

## 4. Discussion

The present study describes the development of a STR genotyping analysis for *W. anomalus*. With the typing scheme, six STR markers with a repeat size of three or six nucleotides were amplified by two multiplex PCRs, M3 and M6, respectively. STR genotyping of 90, mainly Indian *W. anomalus* isolates, resulted in the identification of 38 different genotypes and uncovered hitherto unrecognized outbreak events. WGS SNP analysis of 11 isolates demonstrated similar typing results as those obtained with STR typing, as previously also found for other clinically relevant fungi [38,39,41,57]. With AFST against common antifungals, non-wildtype strains to amphotericin B and azoles were observed for several isolates. One fluconazole non-wildtype isolate harbored an *ERG11* substitution in close proximity to the azole binding site.

### 4.1. W. anomalus Genotyping and Loss of Heterozygosity

The development and application of this *W. anomalus* STR typing assay showed the phylogenetic relationship between 90 isolates. We identified 38 different genotypes, with four large Indian clusters, consisting of five or more isolates, while isolates from other countries were not closely related. To validate the genotypic relations established via STR typing, WGS SNP analysis was performed on three isolates (#7–9) with an identical STR profile and two isolates (#10 and #11) with a nearly identical STR profile. While isolates #8 to #11 differed by eight SNPs at most, they were separated by around 210 SNPs with isolate #7. The examination of these SNPs revealed that 203 SNPs were clustered in approximately 400 kbp of one chromosome and that they were heterozygous present in isolates #8 to #11. This part of the chromosome was homozygous for isolate #7. This difference in zygosity between these isolates is likely due to the loss of one allele, also known as loss of heterozygosity (LOH). LOH continuously occurs in cells, while its rate increases when cells are exposed to high levels of stress, such as exposure to antifungal drugs [58,59]. Previous research demonstrated that fluconazole can induce large LOH events involving partial or complete chromosomes [41,42,43]. As the patients with *W. anomalus* infections were treated with antifungals, the LOH event in the deviant isolate might have been caused by this treatment. LOH is normally repaired via the remaining template chromosome via homologous recombination [60]. The number of SNPs in the residual genome, likely accumulated via single point mutations, is only seven in comparison to the other four isolates, indicating their close relatedness. Of all isolates, genotypes 1 and 2, 5 and 6, 15 and 16, and 19 and 20 differed only in zygosity in one marker. These differences might also be due to LOH. Zygosity differences are also frequently observed in other diploid yeasts such as *Candida tropicalis* and *Pichia kudriavzevii* [40,41,60]. It is very likely that these are caused by LOH, suggesting that this phenomenon is widespread across yeast genera. Previous research on *C. albicans* and *C. tropicalis* demonstrated that LOH increases genetic diversity to rapidly adapt to changing environments by providing fitness advantages and antifungal resistance [61,62,63,64]. All aforementioned isolates were exposed to fluconazole or amphotericin B; however, we did not find a correlation between LOH and susceptibility for antifungals. Altogether, our findings suggest that the described STR scheme can distinguish non-related isolates from those causing an outbreak.

### 4.2. Simultaneous Outbreaks of Different W. anomalus Strains in Single Hospital

Out of the four clusters, two (genotype #5 and #7) were highly related with a single copy number difference in marker M3-a. The genotypic variations with and among the other two clusters were much larger, with differences in at least four STR markers. All these isolates originated from the same hospital, taken from patients admitted in 2018 and 2019, and spread across multiple departments. Thus, in this time frame, there were likely at least three simultaneous outbreaks of different *W. anomalus* strains. To date, outbreaks with *W. anomalus* are rarely described [18,32,33]. Thus, it is remarkable that there were three simultaneous outbreaks in this hospital, suggesting the rather exclusive presence of a certain environmental factor, enriched for *W. anomalus*, within or around the hospital. This is also confirmed by the other 23 genotypes of *W. anomalus* that were found in this hospital. As *W. anomalus* is ubiquitous in the environment, it might be worthwhile to search for the source of introduction within the hospital environment or a shared environmental source [22,65,66].

### 4.3. Antifungal Susceptibility and I469L Substitution in ERG11

AFST against common antifungals for 87 isolates identified two non-wildtype isolates against fluconazole according to CLSI ECVs [54]. It has to be noted that the ECVs are not associated with clinical outcomes. All tested isolates were wildtype against micafungin, and six isolates were non-wildtype against amphotericin B. The two isolates with the highest fluconazole MIC were examined for variations in resistance-associated genes. Two known transcription factors of efflux pumps could not be identified in the *W. anomalus* genome. These factors are likely highly deviant from the orthologous genes in *C. albicans* or might be absent in *W. anomalus*. The isolate with a MIC value of 16 mg/L harbored a novel I469L substitution, while the other isolate did not harbor any variations. Homology modelling of *ERG11* revealed the close proximity of the I469 position to the heme binding site, to which azole derivates bind and inhibit enzyme function. Previously, multiple mutations in the same *ERG11* hotspot region in *C. albicans* have been reported to confer fluconazole resistance [30,67]. Therefore, it is plausible that the I469L substitution alters the azole binding site, and subsequently contributes to the elevated MIC against fluconazole.

In summary, we developed and applied a high-resolution STR typing scheme for *W. anomalus* that is specific, reproducible and fast executable. This assay consists of two multiplex PCRs, which amplify six markers in total. The genotyping of 90 *W. anomalus* isolates identified 38 different genotypes, unveiling four simultaneous outbreak events spread across multiple departments within the same Indian hospital. The comparison of STR and WGS SNP genotyping correlated well, indicating the high resolution of this STR typing scheme. With the construction of an *ERG11* homology model, a potential role of the I469L substitution in fluconazole resistance was suggested.

## Figures and Tables

**Table 1:** Overview of PCR primers for selected STR loci, concentration used in multiplex PCR, details of repeat characteristics and genomic sites.

PCR Panel and Primer Name	Primer Sequence (5′–3′)		Conc ^b^ (µM)	No. of Bases of Primer-Flanking Sequence	Repeat Unit	No. of Repeats ^c^		No. of Alleles	Intragenic/ Locus Protein Coding Gene ^d^
	Forward Primer ^a^	Reverse Primer				Min	Max		
M3									
M3-a	FAM-TCTTGCAAATCGTCAGACATC	ACCATCCTTGTTCCCTTACAAC	5	295	TTG	5	25	16	WICANDRAFT_30999
M3-b	JOE-AGCTTGTAATTGTTGGGCTTG	AGCTCCAGAAACTGAACCAAC	5	128	TGT	6	33	18	WICANDRAFT_34460
M3-c	TAMRA-AGCTCAATTCCAAGCTGAAC	AATCAATCTCTGAGGGTGAAGTC	5	242	ACA	6	33	15	WICANDRAFT_91930
M6									
M6-a	FAM-GTTCGTTTGCAGTTTCTTTCC	ATCACCAACAAACTCGCTACC	2	123	TTCACT	18	38	12	WICANDRAFT_78907
M6-b	JOE-TGCCTTATATGAAGGATGAAGG	AGAGTCACCTCTTGGGCTATG	2	169	GAGAGT	2	19	12	WICANDRAFT_24702
M6-c	TAMRA-CGGAAGGAATAAGAAGCAAAG	GATGTTGGGTATTGTTGTCACTG	10	114	AGCAAC	3	32	15	WICANDRAFT_97047

^a^ FAM, 6-carboxyfluorescein; JOE, 4′,5′-dichloro-2′,7′-dimethoxy-fluorescein; TAMRA, 6-carboxytetramethylrhodamine. ^b^ Concentrations of forward and reverse primers are identical. ^c^ Min, minimum; Max, maximum. ^d^ Reference strain is NRRL Y-366-8 (GCA_001661255.1).

**Table 2:** In vitro AFST metrics for *W. anomalus* (n = 87), according to CLSI M27-S4 standard.

Antifungal	Range (mg/L)	GM (mg/L)	MIC_50_ (mg/L)	MIC_90_ (mg/L)	*n* Non-Wildtype (%)
AMB (n = 87)	0.016–2	0.24	0.25	0.5	6 (6.9)
FLU (n = 87)	0.5–16	1.71	2	4	2 (2.3)
ITC (n = 87)	0.016–1	0.21	0.25	0.5	1 (1.1)
VOR (n = 87)	0.03–0.5	0.09	0.125	0.125	3 (3.4)
ISA (n = 49)	0.031–0.125	0.06	0.063	0.063	N.A.
AFG (n = 48)	≤0.008–0.12	0.04	0.031	0.12	N.A.
MFG (n = 50)	≤0.008–0.032	0.01	0.016	0.031	0 (0)

AMB, amphotericin B; FLU, fluconazole; ITC, itraconazole; VOR, voriconazole; ISA, isavuconazole; AFG, anidulafungin; MFG, micafungin; GM, geometric mean; MIC, minimal inhibitory concentration; N.A., non-applicable.

## Data Availability

All raw read data generated in this study have been submitted to the National Center for Biotechnology Information’s Sequence Read Archive (BioProject ID: PRJNA851035). The coding sequence of *ERG11* determined in this study of isolate 10-11-09-22 was deposited in GenBank under the accession number ON805829.

## Supplementary Materials

The following supporting information can be downloaded at: https://www.mdpi.com/article/10.3390/microorganisms11061525/s1, Figure S1: Minimum-spanning tree of 90 *W. anomalus* isolates marked on isolates date as shown in the color key. Branch lengths indicate the similarity between isolates with thick solid lines (variation in one allele), thin solid lines (variation in two alleles), thin dashed lines (variation in three alleles) and thin dotted lines (variation in four or more alleles). Q, quarter. Figure S2: Alignment of *ERG11* protein sequences of *W. anomalus* (Canpel_ERG11) and *C. albicans* (Canalb_ERG11) as generated with Omega Clustal v.1.2.4. Hotspot regions and the I469 position in *W. anomalus* are marked in yellow and red, respectively. Table S1: Overview of all isolates with MIC values according to broth microdilution methods. MIC values are in mg/L. Table S2: Specificity testing against 14 yeast species.
