# Supplementary material for: Application of Novel Short Tandem Repeat Typing for Wickerhamomyces anomalus Reveals Simultaneous Outbreaks within a Single Hospital
Source: Microorganisms. 2023 Jun 8;11(6):1525. doi: 10.3390/microorganisms11061525 (PMC10303041; doi:10.3390/microorganisms11061525)
Supplement: Supplementary file 1 [file microorganisms-11-01525-s001.zip › microorganisms-2407510-supplementary tables.pdf]

Tabel S1: Overview of all isolates with MIC values according to broth microdilution methods. MIC values are in mg/L.

ID	Alt. ID	Country	City	Hospital Unit	Date of fungemia	Geno-type	AMB	FLU	ITC	VOR	ISA	AFG	MFG
**15525**		India	Chandigarh	Neonatal GE ward	04-08-2019	1	0.25	1	0.12	0.03	NT	0.03	NT
**2064**		India	Chandigarh	Neonatal surgical ICU	25-04-2019	1	0.5	2	0.12	0.03	NT	0.06	NT
**42327**		India	Chandigarh	Pediatric medicine ward	06-03-2019	2	0.25	1	0.25	0.06	NT	0.03	NT
**16119**		India	Chandigarh	Neonatal GE ward	04-08-2019	3	2	4	0.5	0.12	NT	0.06	NT
**40251**		India	Chandigarh	Neonatal ward	16-02-2019	4	1	1	0.5	0.06	NT	0.03	NT
**08-32-01-12**	B-1000	India	Chandigarh			5	0.25	2	0.25	0.125	0.063	NT	0.016
**08-32-01-15**	B-1140	India	Chandigarh			5	0.25	2	0.25	0.125	0.063	NT	<0.008
**08-32-01-16**	B-1148	India	Chandigarh			5	0.25	2	0.125	0.125	0.063	NT	<0.008
**08-32-01-17**	B-1154	India	Chandigarh			5	0.125	2	0.25	0.125	0.125	NT	0.016
**08-32-01-27**	B-1247	India	Chandigarh			5	0.125	2	0.25	0.125	0.063	NT	0.031
**08-32-01-31**	B-1261	India	Chandigarh			5	0.125	2	0.125	0.125	0.063	NT	<0.008
**08-32-01-33**	B-1267	India	Chandigarh			5	0.25	2	0.25	0.125	0.063	NT	0.016
**08-32-01-34**	B-1287	India	Chandigarh			5	0.125	2	0.25	0.125	0.063	NT	0.016
**08-32-01-37**	B-1313	India	Chandigarh			5	0.25	2	0.25	0.125	0.063	NT	0.016
**08-32-01-38**	B-1214	India	Chandigarh			5	0.25	2	0.25	0.125	0.063	NT	0.016
**10415**		India	Chandigarh	Pediatric surgical ICU	28-06-2019	5	0.5	1	0.25	0.12	NT	0.06	NT
**14288**		India	Chandigarh	Neonatal Surgical ICU	29-07-2019	5	0.5	2	0.12	0.03	NT	0.06	NT
**14849**		India	Chandigarh	Pediatric surgical ward	30-06-2019	5	0.5	2	0.25	0.12	NT	0.06	NT
**18454**		India	Chandigarh	Pediatric surgical ward	30-07-2019	5	0.25	4	0.25	0.12	NT	0.06	NT
**41654**		India	Chandigarh	Neonatal ward	07-02-2019	5	0.5	4	0.5	0.12	NT	0.03	NT
**10-04-08-74**	YFJ002-249	Unknown	Unknown			6	0.25	2	0.063	0.125	NT	<0.008	0.016
**34461**		India	Chandigarh	Neonatal surgical ICU	08-01-2019	7	0.5	0.5	0.12	0.03	NT	0.03	NT
**34756**		India	Chandigarh	Neonatal surgical ICU	07-01-2019	7	0.25	1	0.5	0.06	NT	0.12	NT
**35368**		India	Chandigarh	Neonatal ward	18-01-2019	7	1	1	0.25	0.03	NT	0.03	NT
**40659**		India	Chandigarh	Neonatal surgical ICU	20-02-2019	7	1	1	0.5	0.06	NT	0.03	NT
**41522**		India	Chandigarh	Neonatal surgical ICU	01-03-2019	7	0.5	2	0.25	0.06	NT	0.06	NT
**08-32-01-29**	B-1254	India	Chandigarh			8	0.125	4	0.25	0.125	0.063	NT	<0.008
**14278**		India	Chandigarh	Pediatric medicine ward	29-07-2019	9	NT	NT	NT	NT	NT	NT	NT
**19186**		India	Chandigarh	Neonatal surgical ICU	29-08-2019	9	0.25	1	0.12	0.06	NT	0.12	NT

**20676**		India	Chandigarh	CVTS ICU	07-09-2019	9	0.25	1	0.25	0.06	NT	0.06	NT
**21241**		India	Chandigarh	CVTS ICU	07-09-2019	9	NT	NT	NT	NT	NT	NT	NT
**41375**		India	Chandigarh	Neonatal ward	01-03-2019	9	0.5	2	0.25	0.06	NT	0.06	NT
**41755**		India	Chandigarh	Neonatal ward	27-02-2019	9	0.5	1	0.25	0.06	NT	0.06	NT
**41973**		India	Chandigarh	Pediatric medicine ward	19-03-2019	9	0.25	1	0.25	0.06	NT	0.03	NT
**43135**		India	Chandigarh	Pediatric medicine ward	10-03-2019	9	0.5	2	0.25	0.06	NT	0.06	NT
**43696**		India	Chandigarh	Pediatric medicine ward	21-03-2019	9	NT	NT	NT	NT	NT	NT	NT
**41825**		India	Chandigarh	Neonatal ward	04-03-2019	10	0.5	2	0.25	0.06	NT	0.03	NT
**9963**		India	Chandigarh	Neonatal surgical ICU	29-06-2019	11	0.25	1	0.5	0.03	NT	0.03	NT
**15528**		India	Chandigarh	Neonatal GE ward	04-08-2019	12	0.12	2	0.25	0.06	NT	0.03	NT
**34092**		India	Chandigarh	Neonatal ward	27-12-2018	13	0.5	4	1	0.03	NT	0.03	NT
**10-11-08-64**	7385504	The Netherlands	Nijmegen			14	0.125	1	0.125	0.063	0.063	0.016	0.008
**35754**		India	Chandigarh	Neonatal ward	13-01-2019	15	0.5	1	0.25	0.031	NT	0.031	NT
**39100**		India	Chandigarh	Neonatal ward	09-02-2019	16	0.5	2	0.063	0.25	NT	0.063	NT
**39570**		India	Chandigarh	Neonatal ward	15-02-2019	17	1	4	0.5	0.125	NT	0.063	NT
**10-08-05-86**	0268529	The Netherlands	Nijmegen			18	0.125	1	0.125	0.063	0.063	0.016	0.032
**14839**		India	Chandigarh	Neonatal surgical ICU	24-08-2019	19	0.5	4	0.5	0.12	NT	0.03	NT
**36375**		India	Chandigarh	Neonatal ward	16-01-2019	20	0.5	4	0.25	0.12	NT	0.03	NT
**34891**		India	Chandigarh	Neonatal ward	04-01-2019	21	0.25	4	0.25	0.06	NT	0.12	NT
**35347**		India	Chandigarh	Pediatric medicine ward	10-01-2019	22	0.5	2	0.5	0.12	NT	0.06	NT
**34278**		India	Chandigarh	Neonatal surgical ward	06-01-2019	23	0.25	2	0.5	0.06	NT	0.12	NT
**36313**		India	Chandigarh	Neonatal ward	02-01-2019	24	0.5	2	0.5	0.12	NT	0.03	NT
**08-32-01-01**	B-445	India	Chandigarh			25	0.125	2	0.25	0.125	0.063	NT	0.031
**08-32-01-03**	B-539	India	Chandigarh			25	0.25	2	0.25	0.125	0.063	NT	<0.008
**08-32-01-04**	B-641	India	Chandigarh			25	0.063	2	0.5	0.063	0.063	NT	0.031
**08-32-01-05**	B-642	India	Chandigarh			25	0.125	4	0.25	0.125	0.063	NT	0.031
**08-32-01-06**	B-689	India	Chandigarh			25	0.125	4	0.125	0.125	0.063	NT	0.016
**08-32-01-07**	B-749	India	Chandigarh			25	0.25	1	0.25	0.125	0.063	NT	<0.008
**08-32-01-08**	B-781	India	Chandigarh			25	0.25	2	0.25	0.125	0.063	NT	0.016
**08-32-01-09**	B-874	India	Chandigarh			25	0.125	2	0.125	0.125	0.063	NT	<0.008
**08-32-01-10**	B-943	India	Chandigarh			25	0.25	2	0.125	0.125	0.063	NT	0.016
**08-32-01-11**	B-985	India	Chandigarh			25	0.125	4	0.125	0.125	0.063	NT	<0.008

**08-32-01-13**	B-1125	India	Chandigarh			25	0.125	1	0.25	0.125	0.063	NT	0.016
**08-32-01-14**	B-1126	India	Chandigarh			25	0.125	1	0.125	0.125	0.063	NT	<0.008
**08-32-01-18**	B-1157	India	Chandigarh			25	0.125	1	0.125	0.125	0.063	NT	<0.008
**08-32-01-19**	B-1178	India	Chandigarh			25	0.125	1	0.25	0.125	0.063	NT	<0.008
**08-32-01-20**	B-1179	India	Chandigarh			25	0.25	1	0.125	0.125	0.063	NT	<0.008
**08-32-01-21**	B-1198	India	Chandigarh			25	0.25	1	0.25	0.125	0.063	NT	0.016
**08-32-01-22**	B-1199	India	Chandigarh			25	0.125	1	0.25	0.125	0.063	NT	<0.008
**08-32-01-23**	B-1201	India	Chandigarh			25	0.25	1	0.125	0.125	0.063	NT	0.016
**08-32-01-24**	B-1220	India	Chandigarh			25	0.125	1	0.125	0.125	0.063	NT	0.016
**08-32-01-25**	B-1240	India	Chandigarh			25	0.125	2	0.125	0.125	0.063	NT	0.016
**08-32-01-26**	B-1241	India	Chandigarh			25	0.25	1	0.25	0.125	0.063	NT	<0.008
**08-32-01-28**	B-1252	India	Chandigarh			25	0.125	1	0.125	0.125	0.063	NT	0.016
**08-32-01-32**	B-1264	India	Chandigarh			25	0.125	1	0.25	0.125	0.063	NT	0.031
**08-32-01-35**	B-1288	India	Chandigarh			25	0.125	1	0.25	0.125	0.063	NT	0.016
**08-32-01-36**	B-1303	India	Chandigarh			25	0.125	1	0.25	0.125	0.063	NT	0.016
**08-32-01-39**	B-1316	India	Chandigarh			25	0.125	1	0.125	0.125	0.063	NT	0.016
**08-32-01-02**	B-486	India	Chandigarh			26	0.125	1	0.25	0.5	0.063	NT	0.016
**08-32-01-30**	B-1256	India	Chandigarh			27	0.125	1	0.125	0.125	0.063	NT	<0.008
**26329**		India	Chandigarh	Neonatal surgical ICU	19-10-2019	28	0.5	1	0.25	0.063	NT	0.06	NT
**34791**		India	Chandigarh	Neonatal ward	03-01-2019	29	0.5	2	0.5	0.06	NT	0.12	NT
**27043**		India	Chandigarh	Neonatal surgical ICU	25-10-2019	30	0.5	2	0.5	0.12	NT	0.12	NT
**10-08-05-17**	4447266	The Netherlands	Nijmegen			31	0.5	1	0.063	0.063	0.063	0.016	0.016
**10-09-05-80**	CBS 605	Italy	Pavia			32	0.016	2	0.016	0.063	0.031	0.008	<0.008
**10-07-04-90**	6515218	The Netherlands	Nijmegen			33	0.125	1	0.125	0.063	0.032	0.016	0.008
**10-04-14-23**	YFJ002-680	Unknown				34	1	2	0.125	0.125	0.125	<0.008	<0.008
**10-03-01-56**		Qatar				35	0.125	8	0.5	0.25	0.063	0.016	0.016
**10-11-09-22**	4696742	The Netherlands	Nijmegen			36	0.063	16	0.063	0.125	0.063	0.008	<0.008
**10-11-08-65**	8091490585	The Netherlands	Nijmegen			37	0.063	2	0.125	0.063	0.063	<0.008	<0.008
**10-04-06-08**	YFJ002-8	Unknown				38	0.5	4	0.25	0.125	0.125	<0.008	<0.008

AMB, amphotericin B; FLU, fluconazole; ITC, itraconazole; VOR, voriconazole; POS, posaconazole; ISA, isavuconazole; AFG, anidulafungin; MFG, micafungin; NT, not tested; CVTS, cardiovascular and thoracic surgery

Table S2: Specificity testing against 14 yeast species.

Species	M3			M6		
	a	b	c	a	b	c
Candida ablicans	-	-	-	-	-	-
Candida tropicalis	-	-	-	-	-	-
Pichia kudriavzevii	-	-	-	-	-	-
Candida lusitaniae	-	-	-	-	-	-
Candida glabrata	-	-	-	-	-	-
Candida guillermondii	-	-	-	-	-	-
Candida auris	-	-	-	-	-	-
Candida parapsilosis	-	-	-	-	-	-
Candida kefyr	-	-	-	-	-	-
Candida haemulonii	-	-	-	-	-	-
Kodameae ohmeri	-	-	-	-	-	-
Cryptococcus gatti	-	-	-	-	-	-
Cryptococcus neoformans	-	-	-	-	-	-
Saccharomyces cerevisiae	-	-	-	-	-	-
