# Supplementary figures and images for: Application of Novel Short Tandem Repeat Typing for Wickerhamomyces anomalus Reveals Simultaneous Outbreaks within a Single Hospital
Source: Microorganisms. 2023 Jun 8;11(6):1525. doi: 10.3390/microorganisms11061525 (PMC10303041; doi:10.3390/microorganisms11061525)

## Slide 1
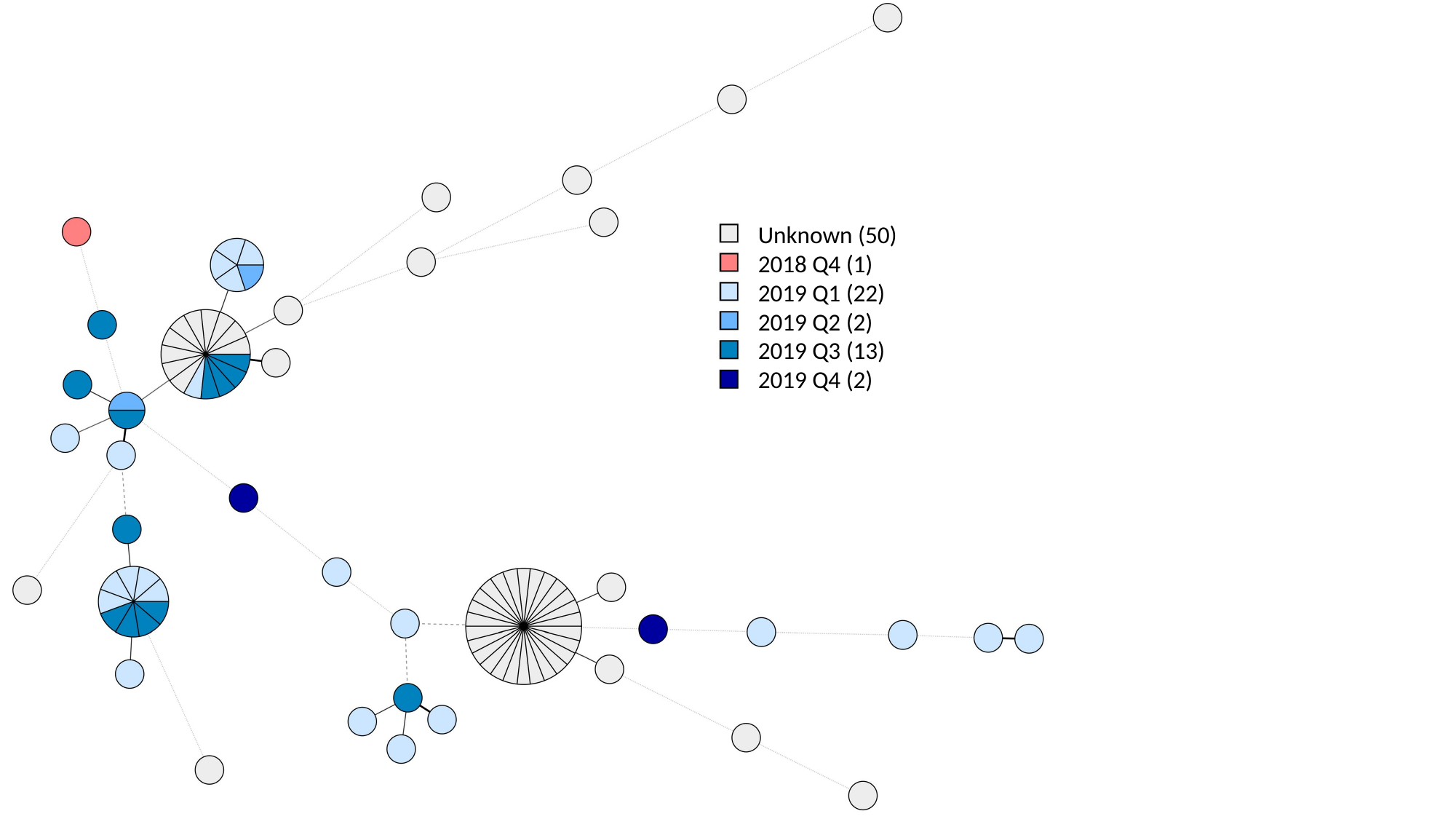

Unknown (50)
2018 Q4 (1)
2019 Q1 (22)
2019 Q2 (2)
2019 Q3 (13)
2019 Q4 (2)

Supplement: Supplementary file 1 [file microorganisms-11-01525-s001.zip › Figure S1.pptx]

## Slide 1
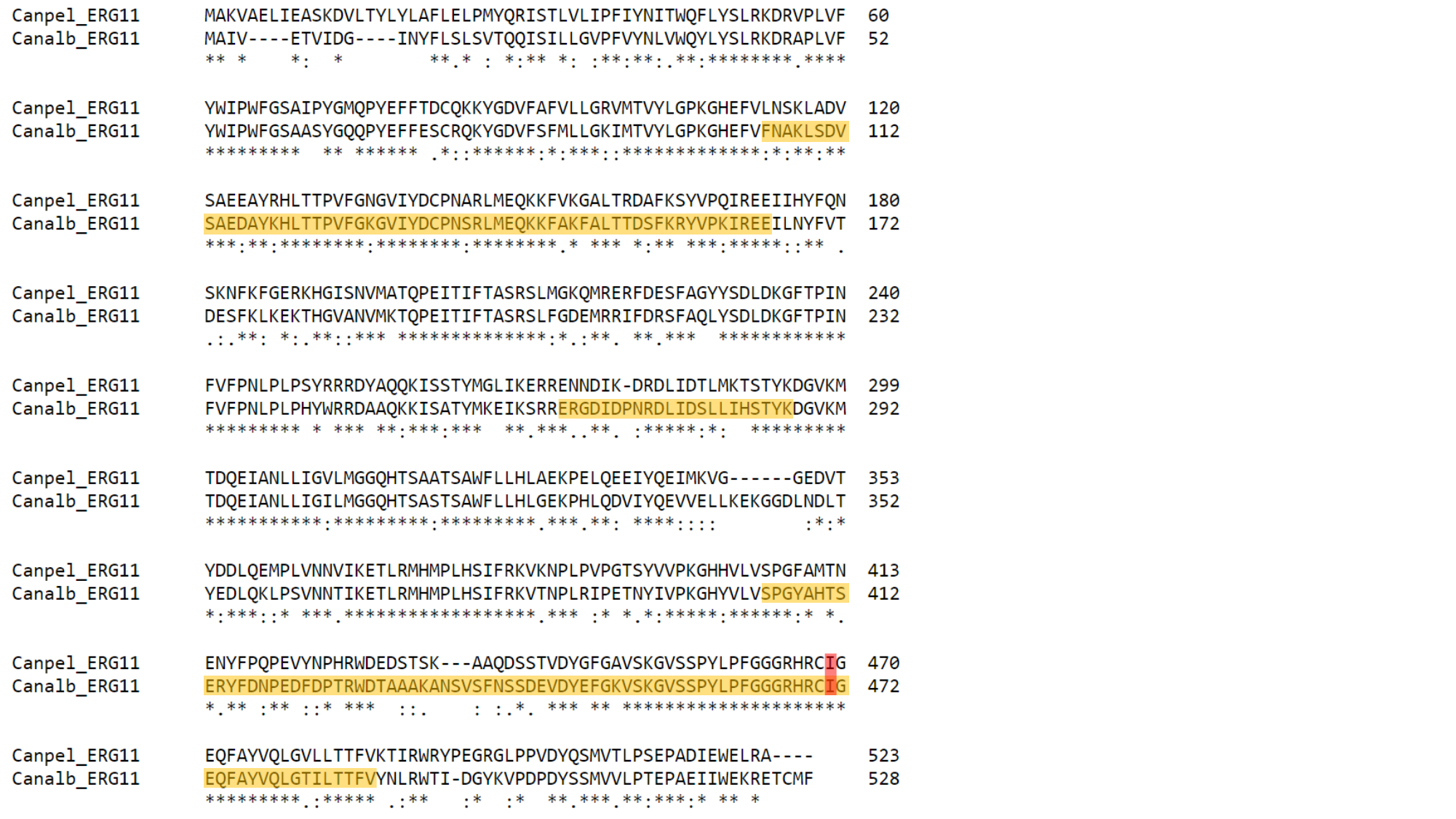

Supplement: Supplementary file 1 [file microorganisms-11-01525-s001.zip › Figure S2.pptx]
